# Coronary Computed Tomography (CT) Angiography as a Predictor of Cardiac and Noncardiac Vascular Events in Asymptomatic Type 2 Diabetics: A 7‐Year Population‐Based Cohort Study

**DOI:** 10.1161/JAHA.116.003226

**Published:** 2016-06-13

**Authors:** David A. Halon, Mali Azencot, Ronen Rubinshtein, Barak Zafrir, Moshe Y. Flugelman, Basil S. Lewis

**Affiliations:** ^1^ Department of Cardiovascular MedicineLady Davis Carmel Medical CenterHaifaIsrael; ^2^ Technion, Israel Institute of TechnologyHaifaIsrael

**Keywords:** computed tomography angiography, coronary disease, diabetes mellitus, plaque, risk stratification, Diabetes, Type 2, Coronary Artery Disease, Computerized Tomography (CT), Primary Prevention, Vascular Disease

## Abstract

**Background:**

Type 2 diabetics are at increased risk for vascular events, but the value of further risk stratification for coronary heart disease (CHD) in asymptomatic subjects is unclear. We examined the added value of coronary computed tomography angiography over clinical risk scores (United Kingdom Prospective Diabetes Study), and coronary artery calcium in a population‐based cohort of asymptomatic type 2 diabetics.

**Methods and Results:**

Subjects (n=630) underwent baseline clinical assessment and computed tomography angiography (64‐slice scanner). Plaque site, volume, calcific content, and arterial remodeling were recorded using dedicated software. Coronary, macrovascular, and microvascular‐related events were assessed over 6.6±0.6 (mean±SD) (range 5.4–7.5) years and all CHD events were adjudicated. Discrimination of CHD events (cardiovascular death, myocardial infarction, unstable angina, or new‐onset angina requiring intervention) (n=41) was improved by addition of total plaque burden to the clinical risk and coronary artery calcium scores combined (C=0.789 versus 0.763, *P*=0.034) and further improved by addition of an angiographic score (C=0.824, *P*=0.021). Independent predictors of a CHD event were United Kingdom Prospective Diabetes Study risk score (hazard ratio 1.3 per 10% 10‐year risk, *P*=0.003) and the angiographic score (hazard ratio 3.2 per quartile, *P*<0.0001). Classification was improved over that by United Kingdom Prospective Diabetes Study and coronary artery calcium scores alone (overall net reclassification improvement 0.24). In subjects with coronary plaque (N=500), mild plaque calcification independently predicted a CHD event (hazard ratio 3.0, *P*=0.02). Computed tomography angiography predicted combined macrovascular but not microvascular‐related events.

**Conclusions:**

Computed tomography angiography provides additional prognostic information in asymptomatic type 2 diabetics not obtainable from clinical risk assessment and coronary artery calcium alone.

## Introduction

Although type 2 diabetes mellitus (DM) is a major risk factor for cardiac and noncardiac vascular events,^1^, ^2^ identification of asymptomatic diabetics at risk for cardiovascular events has proven difficult. Exercise stress testing and myocardial perfusion imaging are not currently recommended for routine screening of diabetics since their use has not led to improved patient outcomes,^3^ and recent guidelines emphasize the need for further evidence to support screening in high‐risk patients with DM.^4^

The coronary artery calcium (CAC) score improves primary risk prediction above that of standard clinical risk predictors both in non‐DM and DM populations^5^, ^6^, ^7^, ^8^, ^9^ and may be useful to select patients who may benefit from more intensive treatment of risk factors.^5^, ^10^ Coronary computed tomography angiography (CTA) can define both calcified and noncalcified coronary plaque, providing a detailed analysis of distribution, extent, and characterization of plaque and has independent value for event prediction.^11^ However, information from detailed CTA analysis in asymptomatic DM without known coronary heart disease (CHD) is limited, particularly over an intermediate or long‐term follow‐up period.^12^, ^13^, ^14^, ^15^

The Lady Davis Carmel Medical Center Diabetic Cohort Study is a prospective, single‐center study of asymptomatic type 2 diabetics with no history of CHD who underwent CAC scoring and CTA and subsequent long‐term clinical follow‐up. We examined the additive value of coronary CTA over CAC and a clinical risk score (United Kingdom Prospective Diabetes Study [UKPDS]) in the prediction of adverse cardiovascular outcomes and developed a model to define a high‐risk subgroup. In addition, since atherosclerosis is a systemic condition with manifestations in diverse vascular beds, particularly so in DM, we examined the relation of coronary arterial findings to noncoronary macro‐ and microvascular‐related events.

## Methods

### Study Population

**Figure 1:**
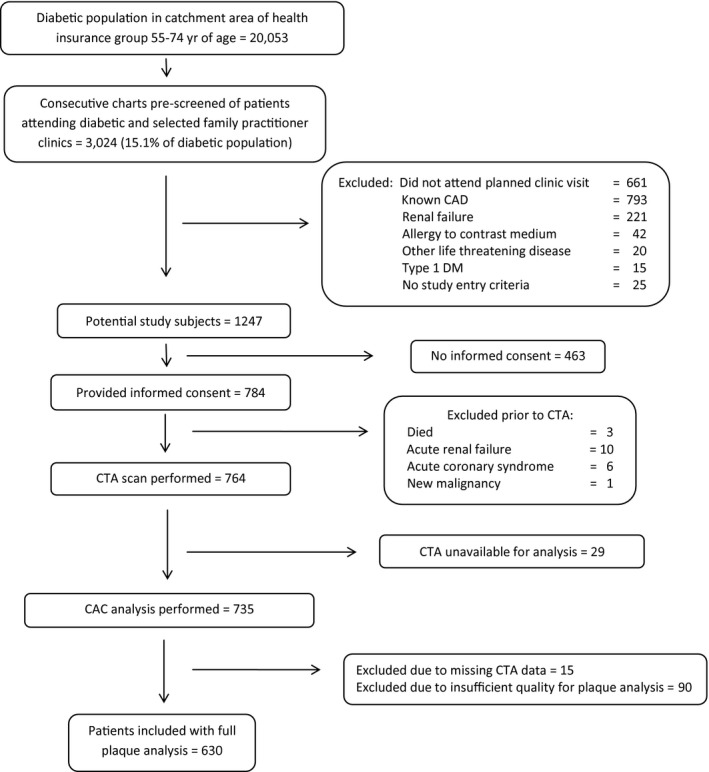
Study population. Derivation of the study cohort from the total population of diabetics in the catchment area of the national health insurance group to which all subjects belonged. CAC indicates coronary artery calcium; CAD, coronary artery disease; CTA, computed tomography angiography; DM, diabetes mellitus.

The study cohort of 630 subjects was derived as detailed in Figure 1. Eligible subjects had type 2 DM, were aged 55 to 74 years, had no history of coronary artery disease (CAD) and at least 1 additional cardiovascular risk factor: DM diagnosed ≥5 years previously; systemic hypertension; current smoking; age >60 years; family history of CHD in a first‐degree relative <55 years; peripheral, cerebral, or carotid vascular disease; and diabetic retinopathy, neuropathy, or albuminuria. Ethics approval was provided by the Ethics Committee of the Lady Davis Carmel Medical Center and all patients provided written informed consent. Exclusion criteria were serum creatinine >1.4 mg/100 mL, allergy to contrast media, and chronic atrial fibrillation. Baseline clinical risk was assessed using the UKPDS CHD risk score. Maximal treadmill exercise was assessed in metabolic equivalents. Treating physicians and study participants received an assessment of risk (below average, average, or above average) based on the CAC score. If high‐grade stenosis of the left main or very proximal left anterior descending arteries was diagnosed, the subject was referred to a single independent cardiologist who assessed the subjects by standard means (generally exercise and/or nuclear stress testing) without specific knowledge of the CTA findings.

### Cardiac Scanning Parameters

CTA was performed between October 2006 and October 2008 with a 64‐channel scanner (Brilliance CT; Philips Healthcare, Cleveland, OH) using a spiral, retrospective, ECG‐gated protocol. β‐Blockers and sublingual nitrates were used routinely. The Agatston CAC score was assessed on a noncontrast‐enhanced scan. A contrast‐enhanced scan was performed with intravenous injection of a bolus of 81±14 mL of contrast (iopromide, 370 mg iodine/mL, Ultravist; Bayer Schering Pharma in 340 [54%] subjects or iohexol, 350 mg iodine/mL, Omnipaque; GE Healthcare, in 290 [46%] patients) at an injection rate of 5–7 mL/s followed by a 50‐mL saline chaser bolus. Mean enhancement of the left main and proximal 25 cross‐sections of the left anterior descending coronary arteries was compared in subjects without coronary plaque for each iodine concentration. Scanning was performed at 120 to 140 kV, effective tube current 500 to 1400 mAs, slice collimation 64×0.625‐mm acquisition, 0.42 seconds gantry rotation time, and pitch 0.2. Reconstruction was performed routinely using a window centered at 75% of the R‐R interval; other windows were used when necessary to minimize cardiac motion. The dose–length product for the axial scan was 779±201 mGy·cm equivalent to 13.2±3.4 mSv.

### Coronary Artery and Plaque Analysis

All scans were examined in axial, multiplanar reformat and short‐axis cross‐sectional views. Window settings were adjusted by the operator to obtain the best differentiation between plaque, surrounding tissue, and vessel lumen and to differentiate between intraplaque densities. Plaque was defined as any extraluminal density that could be clearly assigned to the coronary arterial wall. Plaque position and length were defined along the arterial centerline and related to the respective arterial segment.^16^ Dedicated cardiac analysis software with a plaque analysis application (Cardiac Viewer and Comprehensive Cardiac Analysis, Extended Brilliance Workspace V4.0.2, Philips Healthcare) was used for plaque definition and analysis with manual adjustment as required. Only studies with good or excellent delineation of arterial borders were used for analysis.

Plaque and artery volumes were calculated for each plaque individually as sum of cross‐sectional areas × distance between cross‐sections (pixel spacing) (pixel spacing=field of view/512 and is fixed for each patient). Plaque burden was calculated for each plaque as the volume of plaque divided by the total volume of the same section of coronary artery containing the plaque and total plaque burden as the sum of each individual plaque burden. Area remodeling was measured as maximal cross‐sectional artery area at plaque/plaque‐free cross‐sectional area, sited proximally whenever possible. Bifurcations were assessed by the Medina classification.^17^ Coronary stenosis was assessed visually on a 6‐point scale (0=no plaque, 1=plaque with <25% narrowing, 2=25–49%, 3=50–74%, 4=75–99%, 5=100%). Calcification was assessed visually on a 6‐point scale (0=none, 1=minimal, 2=greater than minimal but <50%, 3=50–70%, 4=71–94%, 5=95–100%). Since proximal plaques may be more event‐prone,^18^ we examined an area–distance index (ADI) calculated as maximal plaque area/distance from aorta for the most proximal plaque and for the plaque with the greatest cross‐sectional area in each of the 3 major and left main coronary arteries. The patient‐based ADI was the sum of ADI from each of the 4 arteries.

The Modified Duke CAD Prognostic Index^19^ is a 6‐point total angiographic score based on the number of plaques, degree of stenosis, and vessel territories involved. The Segment Stenosis Score^19^ is based on a 4‐point score (normal, nonobstructive plaque, moderate stenosis, and severe stenosis) for each coronary segment. The final score is the sum of all segment scores. Segment involvement score is a simple sum of the number of segments with any plaque irrespective of degree of stenosis in each. The Gensini CAD score is calculated from the product of a segment stenosis score (5 grades) and a segmental myocardial weighting factor related to the functional significance of the segment. The final score is the sum of all segmental scores^20^ (Table 1).

**Table 1:** Gensini Coronary Artery Disease Score

SCCT (Modified AHA) Coronary Artery Segment Number^16^	Segment Weighting Factor
1	1
2	1
3	1
4	1
5	5
6	2.5
7	1.5
8	1
9	1
10	0.5
11 nondominant	2.5
11 dominant	3.5
12	1
13 nondominant	1
13 dominant	2
14	0.5
15	1
16	0.5
17	1
18	0.5

Segment Stenosis Grade	Stenosis Score
1% to 24%	1
25% to 49%	2
50% to 69%	4
70% to 99%	8
100%	16

AHA indicates American Heart Association; SCCT, Society of Cardiovascular Computed Tomography.

### Outcome Events

#### Event definition and adjudication

For the primary outcome, CHD events were defined as occurrence of cardiovascular death, nonfatal myocardial infarction (MI), unstable angina, or new‐onset angina pectoris and were examined, according to predefined criteria, by an independent adjudication committee blinded to baseline CTA findings and presented with all clinical and laboratory data. Deaths were identified from a national registry of deaths and details obtained from hospital or family physician records. A diagnosis of MI was based on the presence of 2 of 3 criteria, symptoms, ECG findings, and biomarkers or the presence of new pathological Q waves on the ECG. New‐onset angina was diagnosed only in the presence of definite symptoms requiring revascularization. Hospital discharge reports and laboratory data were obtained from computerized medical records and telephone contact was made if necessary. Record was maintained of diabetic complications or interventions (peripheral vascular, ocular, renal, and diabetic ulcers).

A CHD outcome including only hard events (cardiovascular death or nonfatal MI) was also assessed. A combined noncoronary, vascular outcome included stroke, transient ischemic attack (TIA), carotid or peripheral arterial intervention or amputation, treatment for diabetic ulcer, therapeutic intervention for diabetic retinopathy, or hospitalization for renal failure. Microvascular events included therapeutic intervention for diabetic retinopathy or acute vascular events of the eye or hospitalization for renal failure. A macrovascular event included cardiovascular death, MI, stroke/TIA, carotid arterial intervention, or above‐ankle leg amputation.

### Statistical Analysis

SPSS version 21.0 (SPSS Inc., Chicago, IL), SAS version 9.3 (SAS Institute, Cary, NC), and MedCalc version 15.11.4 (MedCalc Software bvba, Ostend, Belgium) were used for all statistical analyses. Categorical variables are presented as frequencies and continuous variables as mean±SD. Variables were examined with χ^2^ statistic for categorical variables and by Student unpaired *t* test for continuous variables. Interobserver variation was examined in 100 individual coronary plaques in 30 patients following resegmentation. Agreement was assessed by weighted kappa for categorized measures and by intraclass correlation and concordance correlation coefficient for continuous measurements. Time to event was calculated using Kaplan–Meier analysis and results were compared with the log‐rank test. Adjusted Cox proportional hazards models were devised including multivariable stepwise models adjusting for clinical risk scores, CAC score, and CTA variables. Hazard ratios (HR) and 95% CI were calculated from the Cox models. Forward selection Cox regression analysis was employed to define the best CT measures for each category of (1) total plaque extent, (2) angiographic scores, (3) measurements related to a single representative plaque, and (4) plaque characterization. The selected variables from each category were then entered into a forward stepwise multivariate Cox regression model together with the UKPDS CHD score and log_10_(CAC+1). Since plaque characteristics were only examined in subjects with plaque (N=500), there were 2 final models. The first including all patients but without variables describing plaque characteristics (degree of calcification, remodeling, Medina classification, and ADI) and a second model including these plaque descriptors but excluding patients without plaque. Outcome discrimination was assessed with receiver operator characteristic (ROC) curves compared using the method of DeLong.^21^ A 2‐tailed *P*<0.05 was considered statistically significant for all measures. Reclassification was assessed for the CHD outcome prediction model in relation to prediction based on the UKPDS and CAC risk scores alone. The standard 10‐year risk cut‐off values of <10%, 10 to <20%, and ≥20% were adjusted proportionally for the mean study follow‐up period of 6.6 years to assess predicted event rates.^22^, ^23^, ^24^, ^25^ The categorical net reclassification improvement index (NRI) was calculated for 3 risk categories. In addition the continuous NRI was determined which associates any degree of change in predicted risk as consistent or inconsistent with the actual outcome irrespective of any predefined risk categories and the integrated discrimination improvement was assessed (which integrates the NRI over all possible cut‐offs of predicted risk and mathematically corresponds to the difference in discrimination slopes of the 2 models in comparison).^23^ Finally, the predicted CHD event rate was assessed for the upper decile and upper quintile of risk in the study population as defined by the Cox model.

## Results

High‐quality CTA scans suitable for detailed analysis at plaque level were available in 630 patients (Figure 1). Average heart rate during the scan was 60±8 beats per minute (range 39–86 beats per minute). Follow‐up was obtained for all patients. Estimated mean event‐free survival over 7.5 years for the primary CHD outcome was 85.9±0.66% for the study cohort and 83.8±1.9% for patients excluded from the study cohort for technical reasons related to the CTA study (*P*=0.30). Mean enhancement of normal proximal coronary segments was similar for both iodine concentrations used (371±68 versus 359±62 Hounsfield Units, *P*=0.081). There was moderate agreement between observers for most measurements, with weighted kappa for categorized plaque area and volume ranging from 0.5 to 0.625 and concordance correlation coefficient of the continuous measure 0.88. Weighted kappa for CAC grade was 0.53. The weighted kappa for the number of arterial territories with plaque was 0.8 and intraclass correlation was 0.85.

Clinical characteristics and CTA findings overall and for subjects with and without a primary CHD outcome event are presented in total and by sex in Tables 2 and 3. There were sex differences in several baseline characteristics and marked sex differences in plaque extent (Table 3). Univariate predictors of CHD and other outcomes are given in Table 4. Individual outcome events are tabulated in Table 5.

**Table 2:** Baseline Clinical Characteristics

Variable	All	Men	Women	*P* Value	With or without primary endpoint/With or without discreet tabulated variable^a^	*P* Value
N	630	312 (49.5)^b^	318 (50.5)		25 (8.0), 16 (5.0)	0.11
Age, y	63.5±5.3	63.1±5.1	64.0±5.4	0.03	64.8±5.6, 63.4±5.3	0.10
DM, y since diagnosis	10.1±7.5	10.0±7.4	10.2±7.7	0.81	12.9±8.3, 9.9±7.4	0.013
Waist circumference, cm	97.2 (12.5)	98.7±11.5	95.6±13.3	0.002	97.6±9.8, 97.1±12.7	0.81
Insulin treated	138 (21.9)	56 (17.9)	82 (25.8)	0.017	11 (8), 30 (6.1)	0.43
Current smoking	90 (14.3)	55 (17.6)	35 (11)	0.018	7 (7.8), 34 (6.3)	0.60
Past smoking	193 (30.6)	134 (42.9)	59 (18.6)	0.18	16 (8.3), 25 (5.7)	0.23
Pack‐yrs	14.7 (25)	22.2±29.3	7.3±17.2	<0.0001	15.9±21.0, 14.6±25.3	0.74
Hypertension	423 (67.1)	202 (64.7)	221 (69.5)	0.20	25 (5.9), 16 (7.7)	0.39
Family history of CAD	151 (24.0)	67 (21.5)	84 (26.4)	0.15	8 (5.3), 33 (6.9)	0.49
Prior CVA/TIA	39 (6.2)	22 (7.1)	17 (5.4)	0.38	5 (12.8), 36 (6.1)	0.10
Carotid disease/CEA	22 (3.5)	11 (3.5)	11 (3.5)	0.96	4 (18.2), 37 (6.1)	0.024
Ankle/brachial ratio^c^	1.19±0.15	1.20±0.15	1.18±0.14	0.18	1.14±0.12, 1.20±0.13	0.08
Retinopathy	100 (15.9)	46 (14.7)	54 (17.0)	0.44	8 (8), 33 (6.2)	0.51
HbA1c, %	7.4±1.5	7.4±1.5	7.4±1.5	0.76	7.9±1.5, 7.4±1.5	0.025
Creatinine, μmol/L	73.4±16.9	83.1±13.3	65.4±12.4	<0.0001	77.8±17.7, 73.4±15.0	0.075
Cholesterol, mmol/L	4.64±0.93	4.53±0.93	4.77±0.93	0.001	4.93±1.13, 4.63±0.91	0.10
HDL‐C, mmol/L	1.25±0.31	1.13±0.25	1.36±0.33	<0.0001	1.16±0.26, 1.25±0.32	0.07
Triglyceride, mmol/L	1.93±1.39	1.99±1.59	1.59±1.16	0.38	2.85±2.86, 1.88±1.21	0.036
LDL‐C, mmol/L^d^	2.54.0±0.75	2.53±0.75	2.55±0.75	0.69	2.59±0.65, 2.54±0.75	0.65
Cholesterol/HDL ratio	3.9±1.2	4.2±1.3	3.7±1.0	<0.0001	4.5±1.9, 3.9±1.1	0.03
Non‐HDL cholesterol mmol/L	3.39±0.93	3.39±0.93	3.42±0.91	0.84	3.78±1.19, 3.37±0.91	0.042
Albuminuria, μg/mmol creatinine (median, quartiles)	0.90 (0, 2.3)	0.79 (0, 2.4)	0.90 (0, 2.3)	0.27	1.2 (0, 3.4), 1.1 (0, 2.5)	0.82
C‐reactive protein, mmol/L	42.9±52.4	35.2±42.9	49.5±60.0	0.001	47.6±50.5, 41.9±52.4	0.54
Framingham CHD risk score	18.4±9.6	21.7±11.3	15.2±6.2	<0.0001	23.2±11.6, 18.1±9.4	0.008
UKPDS CHD risk score	18.0±11.0	23.6±11.7	12.4±6.5	<0.0001	26.1±16.1, 17.4±10.3	0.001
Aspirin	412 (65.4)	216 (69.2)	196 (61.6)	0.045	32 (7.8), 9 (4.1)	0.08
Clopidogrel	10 (1.6)	7 (2.2)	3 (0.9)	0.19	0 (0), 41 (6.1)	0.40
β‐Blocker	193 (30.6)	75 (24.0)	118 (37.1)	0.0004	14 (7.3), 27 (6.2)	0.61
ACEI/ARB	416 (66.0)	195 (62.5)	221 (69.5)	0.06	24 (5.8), 17 (7.9)	0.31
Calcium channel blocker	143 (22.7)	64 (20.5)	79 (24.8)	0.20	7 (4.9), 34 (7)	0.37
Diuretic	186 (29.5)	80 (25.6)	106 (33.3)	0.03	12 (6.5), 29 (6.5)	0.97
Statin	443 (70.3)	216 (69.2)	227 (71.4)	0.55	32 (7.2), 9 (4.8)	0.26
Maximal treadmill stress, METS^e^	9.1±2.7	10.3±2.6	8.0±2.2	<0.0001	8.0±2.3, 9.2±2.7	0.018
Ischemic ST changes on stress testing^e^	122 (23.4)	73 (28.9)	49 (18.3)	0.004	10 (8.2), 18 (4.5)	0.11

ACEI indicates angiotensin‐converting enzyme inhibitor; ARB, angiotensin receptor blocker; CAD, coronary artery disease; CEA, carotid endarterectomy; CHD, coronary heart disease; CVA, cerebrovascular accident; DM, diabetes mellitus; HbA1c, hemoglobin A1c; HDL‐C, high‐density lipoprotein cholesterol; LDL‐C, low‐density lipoprotein cholesterol; METS, metabolic equivalents; TIA, transient ischemic attack; UKPDS, United Kingdom Prospective Diabetes Study.

a For discrete tabulated variables, the number and percent of individuals with and without the tabulated variable undergoing a primary endpoint event are provided and for continuous tabulated variables the mean and SD of the variable (median and quartiles where stated) for individuals with and without an endpoint event are provided.

b Figures are mean±SD or n (%).

c N=505, values >1.4 excluded due to probable presence of calcified incompressible arteries in 125.

d Calculated LDL‐C values. In 19 patients with triglyceride >400 mg/100 mL LDL‐C values were not calculated.

e N=526, stress testing not performed in 104 subjects mostly for logistical reasons.

**Table 3:** Coronary Artery Atherosclerosis on Coronary CTA

Variable	All^a^	Men	Women	*P* Value (Men vs Women)	With/Without Primary Endpoint^b^	*P* Value (Primary vs no Primary Endpoint)
CAC score, AU (median, quartiles)	58 (1, 333)	164.5 (19, 475)	20.3 (0, 197)	<0.001	52 (0, 308), 389 (128, 706)	<0.001
Coronary artery plaque (any)	500 (79.4)^c^	271 (86.9)	229 (72.0)	<0.001	41 (8.2), 0 (0)	<0.001
None	130 (20.6)	41 (13.1)	89 (28.0)	<0.001	0 (0)	<0.001
1 vessel	113 (17.9)	36 (11.5)	77 (24.2)	<0.001	0 (0)	
2 vessel	154 (24.4)	83 (26.6)	71 (22.3)		9 (5.8)	
3 vessel	233 (37.0)	152 (48.7)	81 (25.5)		32 (13.7)	
LMCA	229 (36.3)	141 (45.2)	88 (27.7)	<0.001	28 (12.2), 13 (3.2)	<0.001
Nonobstructive plaque only	304 (48.3)	140 (44.9)	164 (51.6)	<0.001	11 (3.6), 0 (0)	0.005^d^
1 vessel^e^	100 (15.9)	30 (9.6)	70 (22.0)	<0.028	0 (0)	0.005
2 vessel	111 (17.6)	58 (18.6)	53 (16.7)		6 (5.4)	
3 vessel	93 (14.8)	52 (16.7)	41 (12.9)		5 (5.4)	
LMCA	198 (31.4)	118 (37.8)	80 (25.2)	0.001	6 (5.3), 5 (1.6)	0.031
Nonobstructive plaque in proximal segments (vs no plaque)	269 (42.7)	124 (39.7)	145 (45.6)	0.137	10 (3.7), 1 (0.6)	0.045^d^
Obstructive plaque	196 (31.2)	131 (42.0)	65 (20.4)	<0.001	30 (15.3), 11 (2.5)	<0.001
1 vessel^e^	114 (18.1)	72 (23.1)	42 (13.2)	<0.001	14 (12.3)	<0.001
2 vessel	60 (9.5)	42 (13.5)	18 (5.7)		12 (20.0)	
3 vessel	22 (3.5)	17 (5.4)	5 (22.7)		4 (18.2)	
LMCA	31 (4.9)	23 (7.4)	8 (2.5)	0.005	6 (19.4), 35 (5.8)	0.003
Obstructive plaque in proximal segment	142 (22.5)	94 (30.1)	48 (15.1)	<0.001	26 (18.3), 15 (3.1)	<0.001
Plaques/subject (among those with plaque)^f^	4.5±3.2	5.3±3.4	3.5±2.8	<0.001	6.2±2.6, 4.3±3.3	<0.001
Total plaque length, mm	54.1±49.3	69.0±54.2	36.4±35.6	<0.001	87±42, 51±49	<0.001
Total plaque volume, mm^3^	364.5±393.4	484.2±444.0	222.9±261.1	<0.001	551±307, 348±396	0.001
Total plaque burden^g^	1.8±1.8	2.3±1.9	1.2±1.5	<0.001	3.3±1.6, 1.6±1.8	<0.001
Remodeling index (maximal)	2.2±1.1	2.4±1.2	2.0±0.9	<0.001	2.6±1.1, 2.1±1.1	0.009
Bifurcation involvement (% plaques, N=2241)	1364 (60.7)	583 (51.3)	481 (59.8)	<0.001		
True bifurcation (Medina 1,1,1) (% plaques)	486 (21.7)	324 (22.6)	162 (20.1)	0.39		
Partial bifurcation only (% plaques)	878 (39.2)	559 (38.9)	319 (39.6)			
No bifurcation (% plaques)	877 (39.1)	553 (38.5)	324 (40.2)			
Any partial or true bifurcation (% subjects)	373 (74.6)	209 (77.1)	164 (71.6)	0.16	30 (8.0), 11 (8.7)	0.83
Any true bifurcation (% subjects)	182 (36.4)	117 (43.2)	65 (28.4)	0.001	19 (10.4), 22 (6.9)	0.17
Plaque calcification (% plaques)						
None	204 (9.1)	123 (8.6)	81 (10.1)	0.001		
Minor (Grades 1–2)	527 (23.5)	361 (25.1)	166 (20.6)			
Moderate (Grade 3)	517 (23.1)	353 (24.6)	164 (20.4)			
Heavy (Grades 4–5)	993 (44.3)	599 (41.7)	394 (48.9)			
Subjects with any plaque with mild calcification (vs all other subjects) (% subjects)	293 (58.6)	183 (67.5)	110 (48.0)	<0.001	36 (12.3), 5 (2.4)	<0.001
Degree of stenosis (% plaques)						
0% to 24%	1075 (48)	631 (43.9)	444 (55.2)	0.001		
25% to 49%	828 (36.9)	562 (39.1)	266 (33.0)			
50% to 74%	260 (11.6)	181 (12.6)	79 (9.8)			
75% to 99%	60 (2.7)	49 (3.4)	11 (1.4)			
100%	18 (0.8)	13 (0.9)	5 (0.6)			
Plaque includes inner curve of artery	1665 (75.5)	1054 (74.8)	611 (77.0)	0.25		
Plaque includes outer curve of artery	1206 (54.7)	832 (59.0)	374 (47.1)	<0.001		
Modified Duke CAD prognostic index^h^	2.0±1.3	2.3±1.4	1.7±1.1	<0.001	2.9±1.5, 2.0±1.3	<0.001
Segment stenosis score^i^	5.7±4.1	6.9±4.4	4.3±3.3	<0.001	9.2±4.0, 5.4±4.0	<0.001
Segment involvement score^j^	4.6±2.8	5.4±2.9	3.7±2.5	<0.001	6.6±2.3, 4.4±2.8	<0.001
Gensini CAD score^k^	18.3±16.9	22.7±19.6	13.1±11.1	<0.001	33.4±22.7, 17.0±15.6	<0.001

AU indicates Agatston units; CAC, coronary artery calcium; CAD, coronary artery disease; LMCA, left main coronary artery, CTA, Computed Tomography Angiography.

a For discrete tabulated variables, the number and percent of individuals are provided and for continuous variables, the mean and SD (median and quartiles where stated) are provided.

b Values with and without the tabulated variable for subjects undergoing a primary endpoint event. For continuous variables the mean and SD of the tabulated variable (median and quartiles where stated) for individuals with and without the primary endpoint are provided. Where plaque‐related data rather than subject‐related data are provided, outcome data are not provided.

c Percent subjects.

d *P*‐value for nonobstructive plaque vs no plaque.

e LMCA is counted as 2 vessels and in addition is listed individually.

f All plaque data are calculated from 500 subjects with plaque, 130 subjects with no plaque excluded.

g Total plaque burden was calculated as the sum of the values for each individual plaque.

h The modified Duke prognostic index grades CAD from 0 to 6 according to presence of nonobstructive or obstructive plaque in 1, 2, or 3 coronary arterial territories.^19^

i Segment stenosis score is the sum of the stenosis grades (0–3) from each of 17 coronary arterial segments (theoretical maximum 51).^19^

j Segment involvement score is the number of coronary arterial segments in which any plaque is present (maximum 17).^19^

k The Gensini CAD score (Table 1) is calculated from the multiple of a segment stenosis score (5 grades) and a segment myocardial score related to the functional significance of the segment.^20^

**Table 4:** Clinical and CTA Univariable Outcome Predictors

Variable	Coronary Heart Disease Event^a^		Cardiovascular Death or Myocardial Infarction		Noncoronary Vascular Event^b^		Macrovascular‐Related Event^c^		Microvascular‐Related Event^d^	
	HR (95% CI)	*P* Value	HR (95% CI)	*P* Value	HR (95% CI)	*P* Value	HR (95% CI)	*P* Value	HR (95% CI)	*P* Value
Clinical variables at study entry										
Age (per yr)	1.1 (0.99–1.11)	0.10	1.0 (0.95–1.1)	0.41	1.04 (1.0–1.08)	0.063	1.06 (1.01–1.1)	0.018	1.0 (0.98–1.1)	0.31
Male sex	1.6 (0.87–3.0)	0.13	1.6 (0.64–3.8)	0.33	1.48 (0.98–2.2)	0.06	1.9 (1.2–3.2)	0.009	0.92 (0.55–1.5)	0.74
Duration of DM (per yr)	1.1 (1.01–1.08)	0.012	1.0 (0.98–1.1)	0.29	1.06 (1.04–1.08)	<0.001	1.04 (1.01–1.07)	0.009	1.07 (1.04–1.1)	<0.001
Insulin treated	1.3 (0.66–2.6)	0.446	1.2 (0.43–3.2)	0.76	2.16 (1.4–3.3)	<0.001	1.3 (0.76–2.2)	0.34	2.8 (1.7–4.7)	<0.001
Current smoking	1.2 (0.55–2.8)	0.59	2.0 (0.74–5.6)	0.17	1.25 (0.73–2.1)	0.41	1.4 (0.73–2.5)	0.34	0.80 (0.36–1.8)	0.59
Any smoking	1.7 (0.91–3.1)	0.099	1.6 (0.66–3.9)	0.30	1.0 (0.69–1.5)	0.88	1.4 (0.90–2.3)	0.13	0.79 (0.47–1.3)	0.38
Pack‐yrs (per pack‐yr)	1.00 (0.99–1.01)	0.70	1.0 (0.98–1.0)	0.90	1.0 (0.99–1.0)	0.92	1.0 (0.99–1.01)	0.42	1.1 (0.66–2.0)	0.63
Hypertension	0.76 (0.40–1.4)	0.38	1.1 (0.44–3.0)	0.78	1.05 (0.68–1.6)	0.83	0.87 (0.53–1.4)	0.59	1.2 (0.66–2.0)	0.63
Family history of CAD	0.75 (0.35–1.6)	0.47	1.4 (0.52–3.5)	0.53	0.56 (0.32–0.97)	0.04	0.75 (0.41–1.4)	0.36	0.41 (0.19–0.91)	0.027
Prior CVA/TIA	2.1 (0.83–5.4)	0.12	2.9 (0.85–9.9)	0.089	1.87 (0.97–3.6)	0.06	2.9 (1.5–5.7)	0.002	1.4 (0.58–3.6)	0.44
Carotid stenosis	3.1 (1.1–8.6)	0.033	3.3 (0.77–14.2)	0.11	1.6 (0.63–3.8)	0.33	2.4 (0.95–5.9)	0.064	2.1 (0.78–5.9)	0.14
Retinopathy	1.3 (0.59–2.8)	0.52	1.8 (0.64–4.9)	0.27	4.0 (2.7–6.1)	<0.001	1.7 (0.96–3.0)	0.068	6.0 (3.6–9.9)	<0.001
HbA1c (%)	1.2 (1.0–1.4)	0.028	1.2 (0.92–1.5)	0.21	1.3 (1.2–1.5)	<0.001	1.2 (1.01–1.3)	0.035	1.4 (1.3–1.6)	<0.001
Creatinine clearance (per mL/min)	0.99 (0.98–1.00)	0.16	1.0 (0.98–1.0)	0.95	1.0 (0.99–1.0)	0.49	0.99 (0.98–1.0)	0.094	1.0 (0.99–1.01)	0.81
Total cholesterol (per mmol/L)	1.4 (1.0–1.8)	0.042	0.83 (0.50–1.4)	0.46	1.0 (0.84–1.3)	0.75	1.1 (0.83–1.4)	0.63	1.2 (0.97–1.6)	0.091
HDL–cholesterol (per mmol/L)	0.34 (0.11–1.1)	0.073	0.29 (0.05–1.6)	0.15	0.75 0.48–1.7)	0.75	0.44 (0.18–1.1)	0.065	0.85 (0.37–1.9)	0.69
Cholesterol/HDL‐C ratio (per unit)	1.3 (1.1–1.6)	<0.001	1.1 (0.78–1.5)	0.62	1.1 (0.91–1.2)	0.48	1.2 (1.04–1.4)	0.017	1.18 (1.0–1.4)	0.044
Non‐HDL cholesterol (per mmol/L)	1.5 (1.1–1.9)	0.008	0.94 (0.57–1.5)	0.80	1.0 (0.85–1.3)	0.67	1.2 (0.90–1.5)	0.26	1.3 (0.99–1.6)	0.063
Albuminuria (per quartile)	1.0 (0.79–1.3)	0.85	1.4 (0.94–2.1)	0.096	1.2 (0.99–1.4)	0.06	1.0 (0.85–1.3)	0.70	1.4 (1.1–1.7)	0.009
C‐reactive protein (per quartile)	1.1 (0.86–1.5)	0.38	1.5 (0.99–2.2)	0.057	1.1 (0.89–1.3)	0.52	1.2 (0.95–1.5)	0.14	1.1 (0.85–1.3)	0.57
Framingham CHD risk score (per 10% 10‐yr risk)	1.4 (1.2–1.7)	0.001	1.4 (1.0–1.8)	0.052	1.2 (1.04–1.4)	0.02	1.4 (1.2–1.6)	<0.001	1.2 (1.01–1.5)	0.039
UKPDS CHD risk score (per 10% 10‐y risk)	1.4 (1.3–1.6)	<0.001	1.2 (0.93–1.5)	0.13	1.3 (1.2–1.4)	<0.001	1.4 (1.3–1.6)	<0.001	1.4 (1.2–1.5)	<0.001
Statin therapy	1.5 (0.73–3.2)	0.26	1.3 (0.47–3.5)	0.63	1.1 (0.73–1.8)	0.552	1.7 (0.92–3.0)	0.096	1.1 (0.60–1.9)	0.84
Maximal treadmill stress (per MET achieved)^e^	0.84 (0.72–0.97)	0.017	0.87 (0.70–1.1)	0.22	0.86 (0.79–0.94)	0.001	0.87 (0.78–0.97)	0.011	0.86 (0.78–0.96)	0.008
CTA variables										
CAC score (per quartile)	2.3 (1.6–3.3)	<0.001	2.1 (1.3–2.4)	0.002	1.4 (1.1–1.6)	0.001	2.0 (1.6–2.6)	<0.001	1.1 (0.91–1.4)	0.24
Coronary arteries with plaque (0–3)	4.0 (2.2–7.2)	<0.001	3.1 (1.5–6.2)	0.002	1.4 (1.2–1.8)	<0.001	2.7 (1.9–3.7)	<0.001	1.2 (0.95–1.5)	0.12
Nonobstructive plaque only (vs no plaque) (N=434)	11 vs 0 events^f^	0.039^f^	9 vs 0 events^f^	0.063^f^	1.9 (0.96–3.8)	0.068	11.2 (1.5–82.8)	0.018	1.3 (0.59–2.9)	0.50
No plaque	0 events		0 events							
1 vessel nonobstructive plaque	0 events		0 events							
2 vessel nonobst. plaque (vs none)	6 vs 0 events	0.009	4 vs 0 events	0.044^f^						
3 vessel nonobst. plaque (vs 1–2 v)	3.2 (0.97–10.4)	0.057	3.0 (0.79–11.0)	0.11	1.1 (1.9–3.4)	0.033	2.4 (1.1–5.2)	0.034	1.5 (0.68–3.2)	0.33
LMCA nonobst. plaque (with no stenosis at any site)	3.4 (1.0–11.2)	0.042	3.5 (0.95–13.2)	0.060	1.6 (0.9–2.8)	0.11	3.4 (1.6–7.4)	0.002	0.82 (0.35–1.9)	0.64
LMCA nonobst. plaque (irrespective of other sites)	3.6 (1.8–7.1)	<0.001	5.3 (2.0–13.7)	0.001	1.8 (1.2–2.8)	0.004	3.3 (1.9–5.5)	<0.001	1.2 (0.67–2.0)	0.58
Nonobstructive plaque in proximal segments^g^	6.3 (0.80–49.0)	0.08	5.0 (0.62–39.6)	0.131	1.8 (0.97–3.3)	0.064	7.7 (1.8–32.7)	0.005	1.2 (0.59–2.5)	0.58
Obstructive plaque (any vs all other subjects)	6.6 (3.3–13.1)	<0.001	2.8 (1.2–2.8)	0.022	2.0 (1.4–3.1)	<0.001	3.9 (2.4–6.4)	<0.001	2.04 (1.2–3.4)	0.007
1 vessel (vs no obstructive plaque)	5.2 (2.4–11.5)	<0.001	2.6 (0.92–7.3)	0.071	1.9 (1.2–3.1)	0.010	3.4 (1.9–5.9)	<0.001	2.1 (1.1–3.8)	0.017
2 vessel (vs no obstructive plaque)	8.7 (3.8–19.6)	<0.001	2.5 (0.68–9.3)	0.16	2.3 (1.3–4.1)	0.004	4.8 (2.6–9.1)	<0.001	1.9 (0.89–4.2)	0.097
3 vessel (vs no obstructive plaque)	8.2 (2.6–25.7)	<0.001	4.5 (0.98–21.0)	0.053	2.0 (0.80–5.0)	0.14	4.5 (1.7–11.8)	0.002	2.0 (0.62–6.6)	0.25
LMCA (vs all other subjects)	3.7 (1.6–8.8)	<0.001	1.1 (0.14–7.9)	0.96	1.4 (0.61–3.2)	0.43	2.6 (1.2–5.7)	0.017	2.0 (0.80–5.0)	0.14
Obstructive plaque in a proximal segment (vs all other subjects)	6.6 (3.5–12.4)	<0.001	3.6 (1.5–8.7)	0.004	2.0 (1.3–3.0)	0.002	4.0 (2.5–6.4)	<0.001	1.6 (0.95–2.9)	0.078
All plaques										
Plaques/subject^h^ (per quartile)	2.2 (1.7–2.9)	<0.001	1.6 (1.0–2.6)	0.047	1.22 (1.03–1.4)	0.022	1.7 (1.3–2.2)	<0.001	0.95 (0.75–1.2)	0.68
Total plaque length^h^ (per quartile)	2.6 (1.9–3.6)	<0.001	1.6 (1.1–2.5)	0.026	1.2 (1.05–1.5)	0.012	1.7 (1.3–2.2)	<0.001	0.90 (0.71–1.2)	0.42
Total plaque volume^h^ (per quartile)	2.3 (1.7–3.2)	<0.001	1.5 (0.97–2.2)	0.067	1.25 (1.1–1.5)	0.009	1.6 (1.2–2.0)	<0.001	0.96 (0.75–1.2)	0.77
Total plaque burden^h^ (per quartile)	2.1 (1.5–2.9)	<0.001	1.5 (1.0–2.4)	0.045	1.2 (0.99–1.4)	0.067	1.7 (1.3–2.1)	<0.001	0.99 (0.78–1.3)	0.94
Characteristics of individual plaques										
Maximal plaque X‐sectional area^h^ (per quartile)	1.7 (1.2–2.3)	0.001	1.4 (0.93–2.1)	0.11	1.3 (1.0–1.5)	0.016	1.6 (1.3–2.0)	0.001	1.1 (0.86–1.4)	0.44
Maximal plaque volume^h^ (per quartile)	1.2 (0.89–1.5)	0.27	0.92 (0.62–1.4)	0.69	1.2 (1.0–1.5)	0.047	1.2 (0.93–1.4)	0.21	1.3 (1.0–1.6)	0.050
Maximal plaque burden^h^ (per quartile)	2.0 (1.4–2.7)	<0.001	1.5 (0.97–2.2)	0.068	1.5 (1.2–1.8)	<0.001	1.7 (1.4–2.2)	<0.001	1.3 (1.0–1.7)	0.039
Remodeling index^i^ (per quartile)	1.6 (1.2–2.2)	0.001	1.5 (1.0–2.3)	0.045	1.1 (0.95–1.4)	0.14	1.5 (1.2–1.8)	0.001	0.99 (0.77–1.3)	0.96
True bifurcation^h^^,^ i ^,^ j	1.6 (0.84–2.9)	0.16	1.5 (0.60–3.5)	0.40	1.1 (0.70–1.7)	0.71	1.2 (0.76–2.0)	0.38	1.0 (0.59–1.8)	0.89
Plaque calcification^h^										
None (vs any calcified plaque)^h^	1.3 (0.7–2.5)	0.37	0.77 (0.28–2.1)	0.61	1.3 (0.80–2.0)	0.32	1.1 (0.65–1.8)	0.73	1.0 (0.56–1.88)	0.93
Mild (any plaque CAC grade 1 to 2 vs all others)^h^	5.4 (2.1–13.8)	<0.001	6.5 (1.5–28.1)	0.012	1.5 (0.99–2.2)	0.055	3.1 (1.7–5.8)	<0.001	1.0 (0.59–1.8)	0.90
Moderate/heavy (Grade 3–5 vs none)^h^	0.45 (0.05–4.1)	0.48	2 vs 1 event	1.0^f^	1.0 (0.5–2.0)	0.96	0.55 (0.15–2.0)	0.36	1.0 (0.40–2.5)	0.99
CT angiographic scores										
Modified Duke CAD prognostic index^k^ (0–6 points) (per point)	1.5 (1.3–1.8)	<0.001	1.3 (1.0–1.7)	0.018	1.2 (1.1–1.4)	0.001	1.4 (1.3–1.6)	<0.001	1.2 (1.0–1.4)	0.054
Segment stenosis score^k^ (per quartile)	3.2 (2.2–4.7)	<0.001	2.1 (1.3–3.2)	0.001	1.3 (1.1–1.6)	0.001	2.0 (1.6–2.5)	<0.001	1.1 (0.89–1.4)	0.35
Segment involvement score^k^ (0–16 points) (per quartile)	2.9 (2.0–4.2)	<0.001	1.3 (1.1–1.5)	<0.001	1.2 (1.04–1.5)	0.017	2.0 (1.6–2.5)	<0.001	1.05 (0.84–1.3)	0.68
Gensini CAD score^k^ (per quartile)	3.5 (2.3–5.5)	<0.001	2.3 (1.4–3.7)	0.001	1.4 (1.2–1.7)	<0.001	2.5 (1.9–3.3)	<0.001	1.2 (0.93–1.5)	0.199

CAC indicates coronary artery calcium; CAD, coronary artery disease; CHD, coronary heart disease; CTA, computed tomography angiography; CVA/TIA, cerebrovascular accident or transient ischemic attack; DM, diabetes mellitus; HbA1c, hemoglobin A1c; HDL‐C, high‐density lipoprotein cholesterol; LMCA, left main coronary artery; MET, metabolic equivalent; nonobst, nonobstructive; METS, Maximal treadmill stress; UKPDS, United Kingdom Prospective Diabetic Study; v, vessel.

a Adjudicated coronary heart disease death, myocardial infarction, unstable angina, new‐onset angina.

b Noncoronary macrovascular or microvascular event: Noncoronary vascular death, stroke, transient ischemic attack, carotid or peripheral arterial intervention or amputation, intervention for diabetic retinopathy, hospitalization for renal failure, therapy for diabetic ulcer.

c Cardiovascular death, myocardial infarction, stroke, transient ischemic attack, carotid or peripheral vascular intervention, or amputation of limb.

d Intervention for retinopathy, vascular event of eye, hospitalization for acute renal failure.

e Performed in 526 patients. Exclusions mainly for logistical reasons.

f Regression coefficients do not converge. *P*‐value is from Fisher's exact test from cross‐tabulation.

g Patients with obstructive disease at other sites excluded.

h Among individuals with plaque, N=500.

i For plaque with maximal area remodeling in patient.

j True bifurcation (Medina class 1,1,1) for plaque with largest cross‐sectional area.

k Definitions as for Table 3.

**Table 5:** Individual Outcome Events

Outcome	N (%)^a^
Total mortality	36 (5.7)
Cardiac mortality	8 (1.3)
Cardiovascular mortality	11 (1.7)
MI (nonfatal)	10 (1.6)
STEMI	4 (0.63)
Non‐STEMI	6 (0.95)
Unstable angina	7 (1.1)
New‐onset angina requiring revascularization	20 (3.2)
CVA	20 (3.2)
TIA	9 (1.4)
Revascularization	45 (7.1)
CABG	13 (2.1)
PCI	32 (5.1)
Major amputation	2 (0.31)
Minor amputations	2 (0.31)
Intraocular therapy for retinopathy	52 (8.3)
Acute intraocular vascular event	5 (0.79)
Diabetic ulcer	13 (2.1)
Primary CHD outcome event^b^	41 (6.5)
Cardiovascular death or MI	21 (3.3)
Noncoronary vascular event^b^	96 (15.2)
Macrovascular event^b^	67 (10.6)
Microvascular event^b^	59 (9.4)

CABG indicates coronary artery bypass surgery; CHD, coronary heart disease; CVA, cerebrovascular accident; MI, myocardial infarction; PCI, percutaneous coronary intervention; STEMI, ST elevation myocardial infarction; TIA, transient ischemic attack.

a All fatal events are listed, whereas for other components of the primary CHD outcome (MI, unstable angina, and new chest pain requiring revascularization) the first event (that included in the primary time to event outcome) is listed.

b See Methods in text and notes to Table 3 for definitions.

We referred 24 subjects with high‐grade left main or very proximal left anterior descending coronary artery stenosis for independent assessment by a single physician unaware of the specific CTA findings. In all, 4 of these were referred for invasive angiography and underwent revascularization within 6 months of the CTA scan (1 coronary artery bypass surgery and 3 percutaneous coronary interventions).

### Primary CHD Outcome Predictors

**Figure 2:**
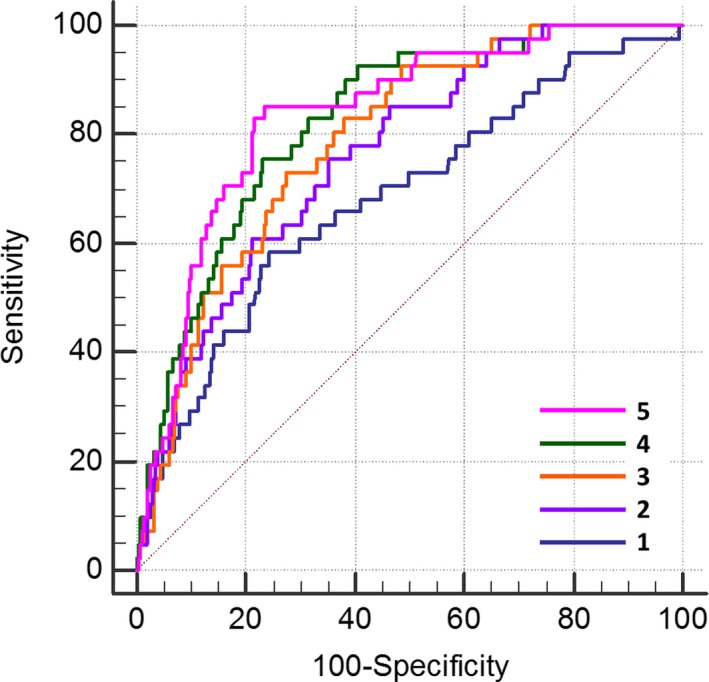
ROC curves for discrimination of a CHD event. The diagonal represents the line of no discrimination. The curves represent the predictive probability of the following: 1. UKPDS CHD risk score alone (C=0.683); 2. the latter plus CAC score (C=0.763, *P*=0.020); 3. the latter plus plaque burden (C=0.789, *P*=0.034); 4. the latter plus the Gensini angiographic score (C=0.824, *P*=0.021); and 5. the latter plus maximal plaque burden and segment involvement score (C=0.836, *P*=0.35) (all *P* values vs immediately previously mentioned ROC curve). Both the CTA‐derived plaque burden and the Gensini score improved outcome discrimination over the combination of the UKPDS CHD risk score and CAC score. CAC indicates coronary artery calcium; CHD, coronary heart disease; CTA, computed tomography angiography; ROC, receiver operator‐characteristic; UKPDS, United Kingdom Prospective Diabetes Study.

**Figure 3:**
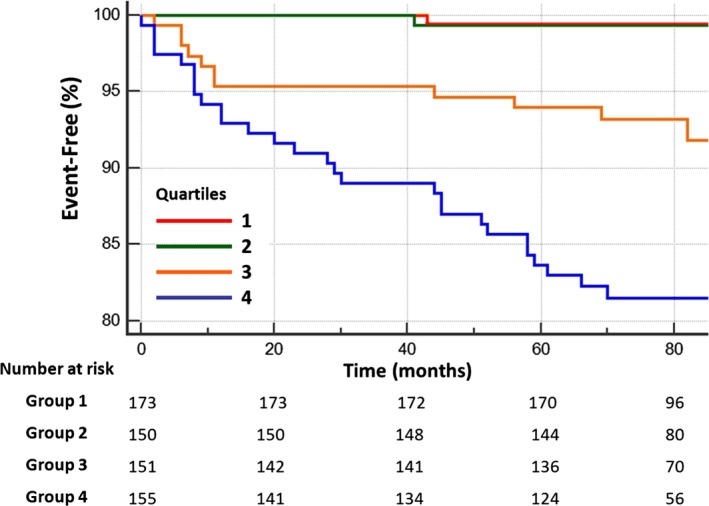
CHD events and the Gensini score. Estimated freedom from a CHD event in relation to quartiles of the CTA‐based Gensini coronary angiography score. (quartile 4 vs 3 *P*=0.006, quartile 3 vs 2 *P*=0.003, quartile 2 vs 1 *P*=0.91). CHD indicates coronary heart disease; CTA, computed tomography angiography.

The UKPDS risk score predicted CHD outcomes (HR 1.44, 95% CI 1.27–1.62, *P*<0.001 per 10% 10‐year risk) with moderate discrimination (C=0.683, 95% CI 0.594–0.772). Components of the UKPDS risk score, including duration of DM, hemoglobin A1c, and cholesterol/HDL‐C ratio, were individually univariable predictors of a CHD event (Table 4). The CAC score predicted outcome independently from the UKPDS risk score (HR 2.1 per quartile, 95% CI 1.5–3.0, *P*<0.001) and when combined with the UKPDS risk score improved the area under the ROC curve (C=0.763, 95% CI 0.694–0.832, *P*=0.02). The area under the curve could be further improved by adding 2 predictors related to extent of coronary plaque (plaque burden) and stenosis (Gensini score) but not by addition of further variables (Table 6 and Figure 2). Tables 3 and 4 show that multiple measures of plaque extent and several characteristics of individual plaques were univariable predictors of the primary CHD outcome. Freedom from CHD events is shown for quartiles of the Gensini score in Figure 3.

**Table 6:** ROC Statistics for Individual Predictors and Combined Predictive Probabilities for All 4 Study Outcomes

Primary CHD Outcome	C‐Statistic (95%CI)	*P* Value
Total cohort (N=630)		
UKPDS risk score (1)	0.683 (0.594–0.772)	0.0001
LogCAC score (2)	0.726 (0.658–0.794)	<0.0001
UKPDS+logCAC scores (3)	0.763 (0.694–0.832)	0.020 vs (1)
UKPDS+logCAC+total plaque burden (4)	0.789 (0.728–0.850)	0.034 vs (3)
4+Gensini score (5)	0.824 (0.768–0.881)	0.021 vs (4)
5+segment involvement score (6)	0.832 (0.778–0.886)	0.35 vs (5)
6+maximal plaque burden (7)	0.836 (0.777–0.894)	0.69 vs (6); 0.27 vs (5)
7+maximal exercise stress (METS)^a^	0.839 (0.769–0.909)	0.48 vs (5)
Patients with plaque (N=500)		
UKPDS risk score (8)	0.655 (0.562–0.747)	0.001
LogCAC score (9)	0.653 (0.570–0.736)	0.001
UKPDS+logCAC score (10)	0.697 (0.610–0.784)	0.177 vs (8)
UKPDS+plaque burden (11)	0.743 (0.670–0.816)	0.114 vs (8)
UKPDS+Gensini score (12)	0.765 (0.725–0.801)	0.001 vs (8)
UKPDS+plaque burden+mild plaque calcification (13)	0.781 (0.742–0.817)	0.002 vs (11)
UKPDS+Gensini+mild plaque calcification (14)	0.786 (0.748–0.821)	0.375 vs (12)
Noncoronary outcomes (N=630)		
UKPDS CHD risk score (15)	0.627 (0.564–0.691)	<0.0001
15+log CAC score (16)	0.650 (0.591–0.709)	0.184 vs (15)
16+total coronary plaque burden	0.650 (0.591–0.701)	0.18 vs (15)
15+albuminuria	0.628 (0.563–0.692)	0.96 vs (15)
15+retinopathy (17)	0.715 (0.655–0.775)	0.0009 vs (15)
17+maximal exercise stress (METS)^a^	0.762 (0.703–0.822)	0.018 vs (17)
Macrovascular outcomes (N=630)		
UKPDS risk score (18)	0.658 (0.584–0.732)	<0.0001
UKPDS+logCAC score (19)	0.745 (0.690–0.801)	0.002 vs (18)
18+prior CVA/TIA (20)	0.756 (0.700–0.812)	0.34 vs (19)
20+plaque burden	0.757 (0.702–0.813)	0.202 vs (19)
20+maximal exercise stress (METS)^a^	0.766 (0.707–0.826)	0.092 vs (19)
Microvascular outcomes (N=630)		
UKPDS CHD risk score (21)	0.669 (0.597–0.740)	<0.0001
21+logCAC	0.669 (0.598–0.741)	0.86 vs (21)
21+retinopathy (22)	0.769 (0.701–0.838)	0.001 vs (21)
22+albuminuria	0.772 (0.703–0.841)	0.36 vs (22)
22+maximal stress (METS)^a^	0.785 (0.712–0.858)	0.018 vs (22)

CAC indicates coronary artery calcium; CHD, coronary artery disease; CVA, cerebrovascular accident; METS, metabolic equivalents; ROC, receiver operator characteristic; TIA, transient ischemic attack; UKPDS, United Kingdom Prospective Diabetes Study.

a For ROC curves including maximal exercise stress N=526.

The addition of CTA data in preliminary Cox regression models showed that among predictors of plaque extent, total plaque burden had the highest HR for a CHD event (*P*<0.001). Among angiographic scores, both the Gensini score (*P*=0.001) and the segmental involvement score (*P*=0.005) were retained in the model. For individual plaques, the maximal plaque burden had the highest HR (*P*<0.001) albeit with very wide confidence intervals. The variables retained in the final 630 subject model were the UKPDS risk score, the Gensini angiographic score, and the total plaque burden, but the latter was excluded from the model when independent variables were entered as quartiles for comparison of HR (Table 7). In a separate model including maximal treadmill exercise, the latter was not an independent CHD outcome event predictor. The combination of UKPDS, log_10_CAC, plaque burden, and the Gensini score provided the best discrimination for a CHD outcome (Figure 2). ROC statistics for individual predictors and combined predictive probability are provided in Table 6.

**Table 7:** Independent Model Predictors of Primary and Secondary Outcomes in Full 630 Patient Cohort

Variable	HR (95% CI)	*P* Value
Primary CHD outcome^a^		
UKPDS (per 10% 10‐year risk)	1.3 (1.1 to 1.5)	0.003
Gensini score (HR per quartile)	3.2 (2.1 to 5.0)	<0.0001
Combined noncoronary vascular outcomes		
UKPDS (per 10% 10‐yr risk)	1.3 (1.2 to 1.4)	<0.0001
Retinopathy at study entry	3.7 (2.5 to 5.7)	<0.0001
Albuminuria (per 10 mg/mmol creatinine)	1.3 (1.0 to 1.6)	0.063
Prior CVA/TIA	1.8 (0.94 to 3.5)	0.075
Macrovascular‐related outcomes		
UKPDS (per 10% 10‐yr risk)	1.3 (1.1 to 1.5)	0.001
Gensini CAD score (per quartile)	2.1 (1.7 to 2.9)	<0.0001
Prior CVA/TIA	2.1 (1.1 to 4.2)	0.03
Microvascular‐related outcomes		
UKPDS (per 10% 10‐yr risk)	1.3 (1.2 to 1.5)	0.0001
Retinopathy at study entry	5.4 (3.2 to 9.0)	<0.0001
Albuminuria (per 100 μg/mg creatinine)	1.04 (1.0 to 1.1)	0.017

CAD indicates coronary artery disease; CHD, coronary heart disease; CVA, cerebrovascular accident; HR, hazard ratio; TIA, transient ischemic attack; UKPDS, United Kingdom Prospective Diabetes Study.

a Maximal plaque burden (HR 17.7 (2.4–131.8), *P*=0.005) was retained in the model when the Gensini score was entered as a continuous variable but was excluded from the model when the Gensini score was entered as quartiles.

### CHD Outcome in Patients With Plaque

**Figure 4:**
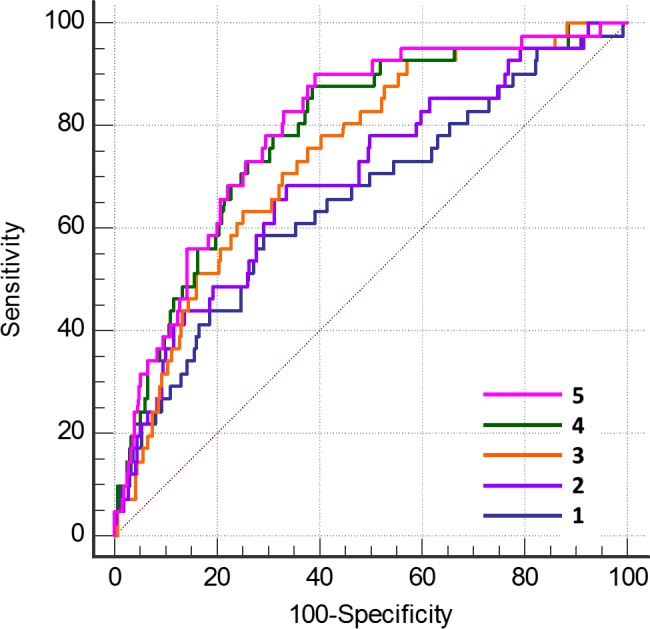
ROC curves for discrimination of a CHD event in patients with coronary artery plaque (N=500). The diagonal represents the line of no discrimination. Addition of each CTA‐derived variable to the combined predictive probability successively improved the C statistic. 1. UKPDS CHD risk score (C=0.655); 2. the latter plus CAC score combined (C=0.697, *P*=0.177); 3. the latter plus plaque burden (C=0.743, *P*=0.035); 4. the latter plus the Gensini angiographic score (C=0.782, *P*=0.040); and 5. the latter plus mild plaque calcification (C=0.798, *P*=0.45 vs 4 above, *P*=0.012 vs 2 above) (all other *P* values vs immediately prior ROC curve). CAC indicates coronary artery calcium; CHD, coronary heart disease; CTA, computed tomography angiography; ROC, receiver operator characteristic; UKPDS, United Kingdom Prospective Diabetes Study.

Plaque characterization was examined for the 500 subjects with coronary artery plaque. Mild calcification was retained in the final model together with the UKPDS and Gensini scores (Table 8). Maximal plaque remodeling, although a univariable CHD outcome predictor (Tables 3 and 4), was not retained in the final model. Total plaque burden was replaced in the model by the Gensini score. The Medina bifurcation status and the ADI ratio were not significant univariable outcome predictors (Tables 3 and 4). On ROC analysis, addition of mild calcification to the combined UKPDS score and total coronary burden significantly increased the AUC, but this was not the case when mild calcification was added to the combined UKPDS and Gensini score (Table 6 and Figure 4).

**Table 8:** Multivariate Cox Model for Prediction of CHD Outcome in Patients With Coronary Plaque (N=500)

Independent Variable	HR	95% CI	*P* Value
UKPDS risk score (per 10% 10‐year risk)	1.3	1.1 to 1.5	0.015
Gensini CAD score (per quartile)	2.5	1.7 to 3.8	<0.0001
Mild (grade 1–2) coronary artery calcification	3.0	1.2 to 7.7	0.02

CAD indicates coronary artery disease; CHD, coronary heart disease; HR, hazard ratio; UKPDS, United Kingdom Prospective Diabetes Study.

### Reclassification

**Figure 5:**
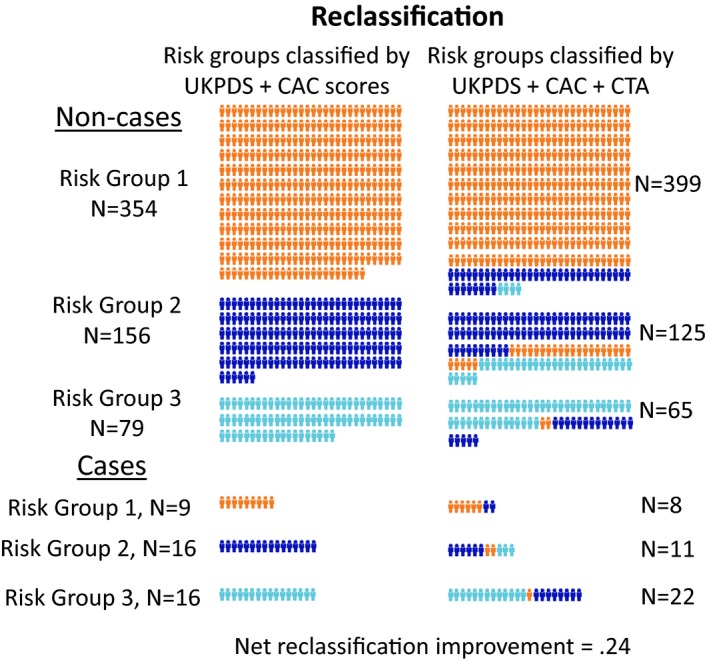
Reclassification of patient risk. The addition of CTA‐derived data to the Cox proportional hazards model improved the classification of primary outcome events over that from the UKPDS and CAC scores alone. Left: classification into low, intermediate, and high risk by UKPDS and CAC scores alone. Right: Classification with the Cox prognostic model based on the clinical risk scores and the CTA findings. Above are subjects with no CHD event (noncases) and below are subjects with a CHD event (cases). Each mannequin represents 1 subject. Among the noncases, 102 subjects (43% of risk groups 2 and 3) were correctly reclassified to a lower risk at the cost of incorrect reclassification of 45 (9% of risk groups 1 and 2) to a higher‐risk group. Among the cases, 11 (44% of risk groups 1 and 2) were correctly reclassified to a higher‐risk at the cost of incorrect reclassification of 5 (16% of risk groups 2 and 3) to a lower‐risk group. The net reclassification improvement was 0.24. CAC indicates coronary artery calcium; CHD, coronary heart disease; CTA, computed tomography angiography; UKPDS, United Kingdom Prospective Diabetes Study.

The category‐based NRI for the CHD outcome is illustrated in Figure 5 and continuous‐event NRI and integrated discrimination improvement are tabulated for both the full and 500‐patient cohort with coronary plaque (Table 9). The data show considerable correct reclassification to higher risk of patients destined to have a primary event with somewhat less correct reclassification to a lower risk of patients not destined to have a primary event. When the subjects without plaque were excluded, extensive correct reclassification of event‐positive patients was at the expense of incorrect reclassification of a large number, but smaller proportion, of subjects not destined to have an event (Table 9).

**Table 9:** NRI and IDI for Final CTA Models Versus Model Including UKPDS and log CAC Score Alone

	Total Cohort (N=630, 41 Events)	Patients With Coronary Plaque (N=500, 41 Events)
Categorical NRI		
Patients with events correctly reclassified to higher risk	11	22
Patients with events incorrectly reclassified to lower risk	5	2
Event net reclassification improvement	14.6%	48.8%
Patients without events correctly reclassified to lower risk	102	54
Patients without events incorrectly reclassified to higher risk	45	150
Nonevent net reclassification improvement	9.6%	−20.9%
Overall net reclassification improvement	0.24	0.28
Continuous NRI		
Patients with events correctly reclassified to higher risk	26	33
Patients with events incorrectly reclassified to lower risk	15	8
Event net reclassification improvement	26.8%	61.0%
Patients without events correctly reclassified to lower risk	402	249
Patients without events incorrectly reclassified to higher risk	187	210
Nonevent net reclassification improvement	36.5%	8.5%
Overall net reclassification improvement	0.632	0.70
Absolute IDI	0.037	0.025
Relative IDI	64.7%	30.3%

CAC indicates coronary artery calcium; CTA, computed tomography angiography; UKPDS, United Kingdom Prospective Diabetes Study; NRI, net Reclassification Improvement; IDI, integrated Discrimination Improvement .

The event rate in the 10% of the population at highest risk for a primary outcome as defined by the survival function of the multivariable Cox model was 27.0% versus 6.5% for the study cohort as a whole. Seventeen of 41 primary outcome events (41.5%) occurred in the upper decile of risk and 68.3% in the upper quintile.

In order to obtain some measure of the potential benefit of risk assessment for subjects found to be in the upper quartile of risk, we have listed the proportion receiving selected preventive medical therapy at baseline and during follow‐up (Table 10). At baseline, only marginally more of the higher‐risk quartile received prophylactic drug therapy than in the study cohort as a whole (compare with Table 2), whereas at follow‐up there was a significant increase in the proportion of high‐risk subjects receiving several of the drugs listed. These changes could be instituted earlier if a risk calculation based on the CTA data was provided to the treating physicians.

**Table 10:** Prevalence of Selected Drug Therapy at Baseline and Late Follow‐Up Among Subjects in the Upper Quartile of Risk for Coronary Heart Disease

Drug	Baseline, N (%)	Follow‐Up, N (%)^a^	*P* Value
Insulin	30 (24.2)	52 (41.9)	<0.0001
Aspirin	99 (79.8)	92 (75.4)	0.011
Clopidogrel	3 (2.4)	14 (11.9)	0.001
β‐Blocker	38 (30.6)	60 (50)	0.0004
ACEI/ARB	87 (70.2)	93 (78.2)	0.005
Ca channel blocker	33 (26.6)	50 (41.0)	<0.0001
Diuretic	40 (32.3)	34 (28.8)	0.029
Statin	94 (75.8)	107 (89.9)	0.15
Ezetimibe	4 (3.2)	6 (5.0)	0.012
Fibrate	18 (14.5)	4 (3.4)	0.44

ACEI, indicates angiotensin‐converting enzyme inhibitor; ARB, angiotensin receptor blocker.

a N varies slightly due to some missing data, particularly in subjects who subsequently died.

### Secondary Outcomes

#### Noncoronary vascular events

Univariable HRs for each of 3 secondary outcomes for individual clinical and CTA variables are presented in Table 4 and the multivariable models in Table 7. The CAC score independently predicted noncoronary vascular events in a preliminary model (CAC, HR 1.25, 95% CI 1.04–1.51 per quartile) but was excluded from the model when retinopathy was added. None of the measures of CAD extent independently predicted noncoronary vascular events. However, in a model including maximal treadmill exercise stress (526 patients), the latter predicted noncoronary vascular events independently of the UKPDS risk score (HR 0.88, 95% CI 0.80–0.96, *P*=0.006). Discrimination was improved by addition of baseline retinopathy to the UKPDS score and more so by addition of maximal exercise stress (Table 6).

#### Combined macrovascular‐related events

Independent predictors in the Cox model were UKPDS CHD risk score, prior stoke or TIA, and the Gensini CAD score (Table 7). Plaque burden had independent predictive value to the UKPDS risk score (*P*<0.0001) but had no independent predictive value in a model including CAC or the Gensini CAD score. When the Gensini score was included in the model, it replaced the CAC score. In a model including maximal treadmill stress, prior stroke or TIA was replaced by maximal stress (HR 0.87, 95% CI 0.78–0.97, *P*=0.01). Discrimination was considerably improved by the addition of CAC to the UKPDS CHD score, but addition of prior CVA/TIA added only minor nominal improvement and addition of plaque burden was of no further value (Table 6). Addition of exercise stress to UKPDS and CAC also added only minimal nominal improvement in discrimination (Table 6).

#### Microvascular‐related events

Univariable predictors are tabulated in Table 4. The UKPDS CHD risk score, retinopathy, and albuminuria at study entry were retained in the multivariable model. No CTA parameters were independent predictors of microvascular events. Maximal exercise tolerance and prior retinopathy improved discrimination (Table 6).

## Discussion

### Primary CHD Outcome

This study showed that in a community‐based cohort of type 2 diabetics with no history of CAD followed for 5.4 to 7.5 years, the extent and location of coronary plaque, as assessed by coronary CTA, had important predictive value for CHD events over and above the UKPDS score and the CAC score. While the CAC score was an important outcome predictor independently of the UKPDS score, the extent of plaque on CTA, a more comprehensive measure of total coronary plaque, was a stronger predictor.

The risk of a CAD event in asymptomatic individuals with DM has been considered equivalent to that of nondiabetics after an acute myocardial infarction.^26^ The findings of the current study clearly confirm that there is a marked heterogeneity of risk among diabetic patients^14^ such that a significant proportion with no or very little coronary plaque are at very low risk and others with more extensive plaque at considerably higher risk for an acute coronary event. In diabetics at low risk, the intensity of preventive medical therapy and frequency of follow‐up may be reduced, particularly when there is intolerance to higher doses of statins or other preventive therapies. The study findings further refine risk assessment among those with coronary plaque by examining in detail the extent of plaque and extent of plaque calcification.

A recent randomized study of screening for CAD with CTA in asymptomatic diabetics (FACTOR 64) did not succeed in showing improvement in outcome when CTA findings, together with recommendations for subsequent medical therapy and revascularization, were provided to the treating physicians.^27^ In that study the control group, without CTA scanning, also received particularly good preventive medical treatment so that differences in therapy between the screened and nonscreened groups were quite small. This is often the case in volunteers for participation in clinical trials, whereas the general population of diabetics may seek and receive less thorough medical care. When randomizing individuals to CTA screened or nonscreened cohorts the screened cohort, as the current study demonstrates, will include diabetics at all levels of risk including many at low risk who have little to gain by more intensive therapy, thus compounding the difficulty to demonstrate utility of generalized screening by CTA in diabetics. Indeed, in the FACTOR 64 study the event rate was only one quarter of that expected at the time of the study size calculation, leading to considerably lower power to detect differences in outcome between the screened and nonscreened cohorts. Thus the FACTOR 64 cohorts, despite an average duration of diabetes mellitus of more than 12 years, randomized individuals at much lower risk for CAD events than originally planned.

While the current study was not designed to test any specific intensive therapy, the identification of a particularly high‐risk cohort should allow a more focused clinical trial of intensive therapy in diabetics in the highest 10% to 20% of risk. A stepwise approach of screening by clinical risk and CAC score followed by CTA in those deemed to be at higher risk would allow characterization of a high‐risk group in whom an intensive preventive regimen directed at lipids, blood pressure, HbA1c, and possibly selective revascularization would be more likely to have clinical benefit, although revascularization may only rarely be indicated.^28^, ^29^, ^30^ Table 10 indicates the potential for early initiation of more comprehensive prophylactic medical therapy in the highest‐risk quartile, by showing increases at late follow‐up in the proportion of subjects receiving selected therapies.

In the current study, a simple scoring system defining site and degree of stenosis (Gensini score) predicted outcome at least as well as more detailed assessments of atherosclerotic burden, leading to improvement in prediction, discrimination, and patient classification. A limited extent of nonobstructive plaque was a relatively benign finding (Tables 4 and 5) in keeping with prior findings in a primarily nondiabetic cohort.^31^ There were no CHD primary outcome events in individuals who had no coronary plaque whatsoever or in those with plaque limited to a single vessel (Table 4).

The importance of CAC in diabetics was demonstrated in a study of 10 377 patients (903 diabetics) that examined mortality over 5 years in relation to CAC in asymptomatic subjects and found a stepwise increase in mortality in diabetics compared to nondiabetics across all CAC categories, while diabetics with low or zero calcium (nearly 40% versus 20.6% in the current study) had an excellent prognosis.^7^ Atherosclerosis has been reported in the absence of calcium (in 6–20% of patients) mostly in symptomatic individuals with a low prevalence of diabetes mellitus.^32^, ^33^, ^34^ In the Coronary Computed Tomography Angiography Evaluation For Clinical Outcomes: An International Multicenter Registry (CONFIRM) study, 12% of individuals with zero calcium had obstructive disease due to noncalcified plaque on CTA.^35^ Coronary calcium is more prevalent in diabetics,^7^ and in the current study there were relatively few patients with purely noncalcific plaque (33, 5.2%, of the total cohort and 22.1% of patients with CAC score zero).

We presented a separate model to examine the contribution of plaque characteristics in patients who had coronary plaque (N=500, 79.4% of subjects). Mild calcification, rather than no calcification or heavier calcification, predicted a CHD event (Table 4), consistent with the previously reported predictive value of spotty calcification in other populations.^36^, ^37^ Although the therapeutic implications of identification of a “vulnerable plaque” phenotype, at least for the individual plaque, are strongly debated,^38^ the predictive value of mild plaque calcification for patient‐based outcomes appears to be a recurring finding.^36^, ^37^ In the study of Kataoka et al^36^ this was a marker for more extensive plaque while in the current study, mild calcification was an independent predictor of a CHD event in the multivariable Cox model (Table 8) and improved somewhat the area under the ROC curve when combined with plaque burden but not when added to the Gensini angiographic score (Table 6). Heavy calcification represents more complex, longer‐established plaque, which has been shown to be more stable.^39^ Analysis of the CT density of the calcified and noncalcified portion of plaques may provide additional information.^40^

In a subanalysis from the CONFIRM registry of 400 asymptomatic diabetics followed for 2.4 years,^12^ multivessel coronary stenosis was a significant predictor of death or MI and when combined with CAC and a clinical risk score was modestly additive to model discrimination (C=0.78 versus 0.74) and improved risk classification. Interestingly, in an analysis from the same registry of a lower‐risk asymptomatic population including ≤15% diabetics, addition of CT parameters to a similar model had minimal additive value.^35^ Thus, CTA may be more relevant when a heightened awareness of clinical risk is appropriate as in diabetics. However, a randomized study of routine screening by cardiac CTA for CAD in DM did not lead to improved outcomes.^27^ Other relatively short‐term studies have reported benefit of CTA in outcome prediction in diabetics.^13^, ^14^, ^41^ The Detection of Ischemia in Asymptomatic Diabetics (DIAD) study randomized patients to myocardial perfusion scintigraphy or no myocardial perfusion scintigraphy to examine the benefit of screening and found no reduction in CHD events in the screened cohort.^42^ In that study, 22% of patients had physiological evidence of CAD at study entry and cardiac death and MI rates were low at 0.6% per year. Similarly in the current study, evidence of ischemia was observed in 23.4% of the 521 patients who underwent treadmill stress testing and the CHD event rate was low at 1% per year and cardiac death or MI at 0.5% per year. In a recent study in asymptomatic diabetics, comparing repeat CTA and myocardial perfusion scintigraphy, ischemia was present in 20% at baseline but resolved in most subjects over 2 years and neither resolution nor new ischemia correlated with CTA parameters.^43^

### Additional Outcomes

The UKPDS risk score and the Gensini CAD score also predicted a combined outcome of coronary or other macrovascular‐related events while only the UKPDS CHD risk score remained a predictor of noncoronary vascular and microvascular‐related events. The latter outcomes were predicted by pre‐existing noncoronary macro‐ or microvascular disease (prior stroke/TIA, retinopathy, and albuminuria). Thus, while the current findings portray a close relationship between coronary CTA findings and CHD events, they demonstrate a lack of association with events related to other vascular beds.

### Limitations and Advantages

This was a single‐center analysis. However, the study population was derived from multiple diabetic and family practitioner clinics based on a large population of more than 20 000 diabetic patients in the age group studied who were under routine medical care and follow‐up. There are presently only limited data of this kind in a diabetic cohort of similar size and follow‐up. The primary event rate was lower than originally expected for a diabetic cohort, a finding previously noted in prospective studies of unselected asymptomatic diabetics.^42^ Detailed follow‐up was available from all national Health Maintenance Organizations and hospitalizations, although in the very few patients no longer resident in the country some data might have been overlooked despite telephone contact. This study was limited to whites, whereas CAC scores differ between ethnic groups and doubling of the score increased the HR for coronary events somewhat more in blacks and Chinese than in whites.^44^ We included unstable and new‐onset angina pectoris in the primary outcome but only after careful adjudication, and in the case of the latter only if it led to a revascularization procedure. Interobserver variation in plaque assessment was considerable, related at least in part to limited spatial resolution, limited contrast differentiation, and calcium artifact. This may improve with newer scanners and requires further investigation. Maximal exercise stress was not available for logistic reasons in the later stages of patient recruitment (104 patients), leading to a lower number of outcome events when this variable was included in some of the outcome prediction models and ROC analysis. The study was not designed to define treatment or to show benefit of routine screening on clinical outcomes.

## Summary and Clinical Implications

The study demonstrates that asymptomatic type 2 diabetics can be stratified by clinical and noninvasive testing into very low and successively higher risk cohorts for cardiac and other vascular events. The low overall cardiac event rates (1% per year for the primary CAD outcome) represent those seen in the current era in a well‐treated asymptomatic diabetic cohort, despite widely prevalent coronary atheroma on CTA and ECG criteria for stress‐induced ischemia in 23%. Diabetics with no coronary plaque (20% of the study cohort) are at extremely low risk for a CHD event. Importantly, the low event rate implies that large patient populations will need to be studied in order to demonstrate further preventive or therapeutic advances. In the present study, 10% of the overall study population was identified with a CHD event rate >4 times higher than that of the general study population. Selection of such a cohort for future studies might be helpful. However, although the CHD event rate was 4 times higher in the upper decile of risk, intervention in this high‐risk decile could be beneficial in only the 41.5% of all primary CHD outcomes occurring in this selected subset. Preventive or therapeutic intervention is likely to be more effective in the highest‐risk quintile in which 68.3% of all CHD events occurred.

## Sources of Funding

The study was supported by a research grant from the European Foundation for the Study of Diabetes.

## Disclosures

None.
